# Supplementary material for: Rough Fabry–Perot cavity: a vastly multi-scale numerical problem
Source: Nanophotonics. 2025 Nov 19;14(27):5375–84. doi: 10.1515/nanoph-2025-0448 (PMC12717928; doi:10.1515/nanoph-2025-0448)
Supplement: Supplementary file 1 — Supplementary Material Details [file j_nanoph-2025-0448_suppl_001.docx]

**Supplementary Information**

Rough Fabry-Perot cavity: a vastly multi-scale numerical problem

Tetiana Slipchenko,^#^ Jaime Abad-Arredondo,^#^* Antonio Consoli, Francisco J García Vidal, Antonio I Fernández-Domínguez, Pedro David García, Cefe López*

^1^Instituto de Ciencia de Materiales de Madrid (ICMM), Consejo Superior de Investigaciones Científicas (CSIC), Calle Sor Juana Inés de la Cruz, 3, 28049 Madrid, Spain.

^2^Dep. Física Teórica de la materia Condensada, Universidad Autónoma de Madrid, Tomás y Valiente, 28049 Madrid and IFIMAC

^3^Escuela de Ingeniería de Fuenlabrada (EIF), Universidad Rey Juan Carlos (URJC), Camino del Molino 5, 28942 Fuenlabrada, Madrid, Spain.

† [jaime.abad@uam.es](mailto:jaime.abad@uam.es); § c.lopez@csic.es

1. Model for diffuse FP cavities

Single reflection

In this document, we attempt to find an analytical model to describe the effect of roughness on the FP resonances. The key assumption is that a plane wave, upon impinging on a rough surface will be reflected (and transmitted) as a superposition of waves with slightly different phase offsets, so that: $r\to r \Delta\phi$, where $\Delta\phi=\sum_{\phi} f\left( \phi\right)e^{i\phi}$. We assume that this distribution of phase offsets is continuous, and that it follows a normal distribution such that [1]:

$$\begin{aligned} \Delta\phi=\sum_{\phi} f\left( \phi\right)e^{i\phi}\Rightarrow\frac{1}{\sigma_{\phi}\sqrt{2\pi}}\int d\phi e^{-\frac{1}{2}\left( \frac{\phi}{\sigma_{\phi}} \right)^{2}} e^{i\phi}=e^{-\frac{1}{2}\sigma_{\phi}^{2}}. \#\left( S1 \right) \end{aligned}$$

This shows that each time a plane wave interacts with the rough surface, the phase superposition leads to an exponential attenuation, similar to the effect of losses. We can furthermore parametrize $\sigma_{\phi}$ in terms of the geometrical step height distribution of the edge as: $\sigma_{\phi}=\alpha k\sigma_{h}$, where $\alpha$ is a scaling factor, $k=n_{s}k_{0}$ is light’s wavevector in the cavity and $\sigma_{h}$ is the height standard deviation. Furthermore, as shown in the simulations of the main text, this reflectance loss saturates, so that the reflectance factor at the rough mirror is replaced by:

$$\begin{aligned} r^{'}\equiv\left( r-r_{0} \right)e^{-\frac{1}{2}\left( \alpha k\sigma_{h} \right)^{2}}+r_{0}\#\left( S2 \right) \end{aligned}$$

Where $r$ is the pristine case reflectance, and $r_{0}$ is the roughness-saturated value.

Multiple Scattering problem

We now use this to set up a multiple scattering problem to see how this affects the FP modes. We assume that a plane wave of free space wavevector $k_{0}$ impinges on the mirror of transmission and reflection coeficients: $t$, $r,$ and travels in the cavity of length $L$. It then scatters with the roughened mirror and transmits. We assume the phase superposition is only imparted in reflection. Nevertheless, this will not affect the resonant modes of the diffuse cavity. The total light field at the end of the cavity is:

$$\begin{aligned} E=E_{0}te^{ikL}t\sum_{n} \left( \sqrt{rr^{'}}e^{ikL} \right)^{2n}=\frac{E_{0}t^{2}e^{ikL}}{1-\left( \sqrt{rr^{'}}e^{ikL} \right)^{2}},\#\left( S3 \right) \end{aligned}$$

and therefore, the intensity at the end of the FP is

$$\begin{aligned} I\propto EE^{*}=\frac{\left| E_{0} \right|^{2}\left| t \right|^{4}e^{i\left( k-k^{*} \right)L}}{\left[ 1-\left( \sqrt{rr^{'}}e^{ikL} \right)^{2} \right]\left[ 1-\left( \sqrt{r^{*}\left( r^{'} \right)^{*}}e^{-ik^{*}L} \right)^{2} \right]}.\#\left( S4 \right) \end{aligned}$$

This can be rewritten by performing the assignment $\sqrt{rr^{'}}e^{ikL}\equiv e^{O}$, where:

$$\begin{aligned} O=ikL+\frac{1}{2}\ln\left( r \right)+\frac{1}{2}\ln\left[ r_{0}+\left( r-r_{0} \right)e^{-\frac{1}{2}\left( \sigma_{\phi} \right)^{2}} \right],\#\left( S5 \right) \end{aligned}$$

the intensity can be written in the compact form

$$\begin{aligned} I\propto\frac{\left| E_{0} \right|^{2}\left| t \right|^{4}}{2}\frac{e^{-2n_{s}^{''}k_{0}L}e^{-2O^{'}}}{\cosh\left( 2O^{'} \right)-\cos\left( 2O^{''} \right)}.\#\left( S6 \right) \end{aligned}$$

where *n_s_*′ and *n_s_*′′ are the real and imaginary parts of the refractive index of the slab material.

Splitting $O$ into its real and imaginary parts by assuming $\left\{ r,r_{0} \right\}\mathbb{\in R}$, one has

$$\begin{aligned} O=\left\{ -n_{s}^{''}k_{0}L+\frac{1}{2}\ln\left( r \right)+\frac{1}{2}\ln\left[ r_{0}+\left( r-r_{0} \right)e^{-\frac{1}{2}\left( \sigma_{\phi} \right)^{2}} \right] \right\}+i\left[ n_{s}^{'}k_{0} L \right].\#\left( S7 \right) \end{aligned}$$

Resonances will take place when $O^{''}=\pi n$, where $n$ is an integer that will be used to index the resonances, with corresponding resonant frequencies $\omega_{0,n}=\frac{\pi nc}{n_{s}^{'}L}$. Expanding the frequency around these resonances as $\omega_{n}=\omega_{0,n}+\delta\omega$, defining $\Delta\omega=\pi c/n_{s}^{'}L$, and the loss tangent as $\chi\equiv n_{s}^{''}/n_{s}^{'}$, the intensity fulfils

$$\begin{aligned} I\propto\frac{1}{\cosh\left( -2\pi\chi\left[ n+\frac{\delta\omega}{\Delta\omega} \right]+\ln\left( r \right)+\ln\left[ r_{0}+\left( r-r_{0} \right)e^{-\frac{1}{2}\left( \sigma_{\phi} \right)^{2}} \right] \right)-\cos\left( 2\pi\frac{\delta\omega}{\Delta\omega} \right)}.\#\left( S8 \right) \end{aligned}$$

For the large cavities under study, $n$ will be large, and therefore, we can assume $\delta\omega\ll n \Delta\omega=\frac{n\pi c}{n_{s}^{'}L}$. Taylor expanding the cosine around $\delta\omega\approx0$, the intensity acquires a Lorentzian profile:

$$\begin{aligned} I\propto\frac{1}{\left[ \cosh\left( -2\pi\chi n+\ln\left( r \right)+\ln\left[ r_{0}+\left( r-r_{0} \right)e^{-\frac{1}{2}\left( \sigma_{\phi} \right)^{2}} \right] \right)-1 \right]+\left( 2\pi\frac{\delta\omega}{\Delta\omega} \right)^{2}}.\#\left( S9 \right) \end{aligned}$$

From this expression we obtain the quality factor of the resonance, given by

$$\begin{aligned} Q_{n}=\frac{\sqrt{2}n\pi}{\sqrt{\cosh\left( -2\pi\chi n+2\ln\left( r \right)+\ln\left[ \frac{r_{0}}{r}+\left( 1-\frac{r_{0}}{r} \right)e^{-\frac{1}{2}\left( \sigma_{\phi} \right)^{2}} \right] \right)-1}}\#\left( S10 \right) \end{aligned}$$

In absence of roughness, $\sigma_{\phi}=0$, this expression leads to the *pristine* cavity quality factor

$$\begin{aligned} Q_{n}^{\left( P \right)}=\frac{\sqrt{2}n\pi}{\sqrt{\cosh\left( 2\ln\left( r \right)-2\pi\chi n \right)-1}}.\#\left( S11 \right) \end{aligned}$$

Grouping all the broadening mechanisms beyond roughness into the same variable, $\Gamma_{n}\equiv-2\ln\left| r \right|+2\pi n\chi$, and defining a variable that contains the reflectivity drop due to roughness as $\eta\equiv\beta+\left( 1-\beta\right)e^{-\frac{1}{2}\left( \sigma_{\phi} \right)^{2}}$, with $\beta\equiv r_{0}/r,$ then the quality factor can be written as

$$\begin{aligned} \left( \frac{1}{Q_{n}} \right)^{2}-\left( \frac{1}{Q_{n}^{\left( P \right)}} \right)^{2}=\frac{\cosh\left( \ln\left[ \eta\right]-\Gamma_{n} \right)-\cosh\left( \Gamma_{n} \right)}{2n^{2}\pi^{2}}.\#\left( S12 \right) \end{aligned}$$

Defining $A_{n}\equiv\frac{\sqrt{2}n\pi}{Q_{n}^{\left( P \right)}}$ for compactness, one can see that

$$\begin{aligned} \cosh\left( \Gamma_{n} \right)=1+\left( \frac{\sqrt{2}n\pi}{Q_{n}^{\left( P \right)}} \right)^{2}=1+A_{n}^{2}\#\left( S13 \right) \end{aligned}$$

and finally, the quality factor can be written in a compact form as

$$\begin{aligned} Q_{n}=\frac{Q_{n}^{\left( P \right)}}{\sqrt{1+\frac{\left( 1-\eta\right)}{2\eta}\left[ F_{n}^{(+)}-F_{n}^{\left( - \right)}\eta\right]}}.\#\left( S14 \right) \end{aligned}$$

Where we have introduced the parameters $F_{n}^{(\pm)}\equiv\frac{\left[ A_{n}^{2}+1 \right]}{A_{n}^{2}}\pm\sqrt{\frac{\left( A_{n}^{2}+2 \right)}{A_{n}^{2}}}$. This constitutes the main result of this section: an analytical expression for the behavior of the quality factor of a Fabry-Perot (FP) mode as a function of roughness. We recap the definitions of the relevant magnitudes in the expression:

$$\begin{aligned} \beta\equiv\frac{r_{0}}{r},\#\left( S15a \right) \end{aligned}$$

$$\begin{aligned} \eta=e^{-\frac{\sigma_{\phi}^{2}}{4}}\left[ \cosh\left( \frac{\sigma_{\phi}^{2}}{4} \right)-\sinh\left( \frac{\sigma_{\phi}^{2}}{4} \right)\left( 1-2\beta\right) \right],\#\left( S15b \right) \end{aligned}$$

$$\begin{aligned} A_{n}\equiv\frac{\sqrt{2}n\pi}{Q_{n}^{\left( P \right)}}.\#\left( S15c \right) \end{aligned}$$

By relating the phase offset’s standard deviation to the RMS geometric roughness, we have the complete expression $\sigma_{\phi}=\alpha k\sigma_{h}$. Where $\alpha$ is a geometric sensitivity factor, $k=n_{s}k_{0}$ is light’s wavevector in the cavity and $\sigma_{h}$ is the RMS roughness. Then, this expression provides several fitting parameters with significant physical meaning:

- $Q_{n}^{\left( P \right)}$: Representing the pristine case quality factor.
- $A_{n}$: capturing the mode’s loss channels in the pristine case and therefore set the level at which the roughness-induced loss levels become relevant.
- $\kappa$: capturing the sensitivity of the modes to roughness.
- $\beta$: capturing the saturating behavior of the quality factor.

As a final remark, since $\eta\approx\beta$ in the large roughness limit, this expression predicts that the quality factor saturates to the value

$$\begin{aligned} Q_{n}^{\left( sat \right)}=\frac{Q_{n}^{\left( P \right)}}{\sqrt{1+\frac{\left( 1-\beta\right)}{2\beta}\left[ F_{n}^{(+)}-F_{n}^{(-)}\beta\right]}},\#\left( S16 \right) \end{aligned}$$

reproducing the numerical simulation’s behavior.

1. Numerical Methods

To investigate the resonant modes and frequency characteristics of a passive FP cavity, we performed eigenfrequency simulations using the Electromagnetic Waves, Frequency Domain interface within COMSOL Multiphysics.

The computational domain is composed of two regions: a central rectangular domain with a width of *W* = 10 μm, a variable length, $L$, ranging from *L* = 30 μm to *L* = 250 μm, and a refractive index of *n_s_* = 3.86; and an outer air domain terminated by scattering boundary conditions. To introduce boundary roughness, we follow two complementary approaches:

- On the small cavity calculations, we implement a random boundary edge with a given RMS displacement, and correlation length of 100 nm by the method of wave addition with a normal distribution of amplitudes, and a uniform phase offset distribution. This results on a highly oscillatory profile, which demands a high density of mesh elements in the rough edge to properly resolve the eigenmode’s profile and linewidths.
- On the large cavity simulations, to save on computational cost to be able of increasing the cavity length to the 250 $\mu$m limit, the smooth corrugation method is substituted by a simple crenelated profile consisting of *N*_sect_ sections of different length (See 1d). These sections have the same width but vary in height, and their number is adjustable to control the degree of surface irregularity. The height of each section is uniformly distributed with height h.

For the eigenfrequency analysis, we specified an eigenvalue search range centred around 500 THz. This setup provides insights into the cavity’s mode structure and frequency response under different levels of boundary roughness.

1. Soft-edged cavities: graded index confinement

The results presented in the main text are obtained from two complementary theoretical approaches: numerical simulations of 2d rectangular FP cavities with varying degrees of roughness, and an analytical model derived from a 1d FP cavity. In both of these cavities, the optical confinement stems from refractive index contrast between the material medium of the cavity, and the external medium. Nevertheless, in previous realizations of diffuse FP cavities from laser diodes (see Fig.1a in main text), the lasing region is basically defined by the gain region, delimited by the pumping electrodes. Therefore, optical confinement is provided by the electrode, and the little refractive index contrast that gating produces in the material through injection. The spectra of these high-power laser diodes, with lengths of ~360 $\mu$m, measured below threshold may range from clearly resolved resonances, to almost continuum spectra for broader lasing region lasers, where no discrete resonances may be observed.

To demonstrate the effect of the loss of lateral confinement, we implement a soft-edge cavity model in which the refractive index profile of the waveguide smoothly varies between the refractive index of the environment, and that of the waveguide, similar to graded index waveguides. This mimics the gradient of refractive index introduced by injection from the electrode. In **Fig. S1** (a,b) we show the resonant frequencies obtained for a refractive index contrast of 2, and 0.2 obtained from an eigenvalue calculation in COMSOL. This simulation returns many modes that have most the electro-magnetic energy outside of the cavity, and thus represent spurious modes that arise from the implementation of the external boundary conditions representing an open domain (scattering boundary conditions in our case). To distinguish between physical and spurious modes, we plot the fraction of EM energy inside the cavity for the different eigenmodes, and filter these modes by establishing a threshold value of minimal energy inside the cavity. This allows to find that the main difference between Fig. S1 (a) and Fig. S1 (b) is a reduction of the free-spectral range in the cavity with less refractive index contrast.

We also attempt to study the effect of roughness in these cavities through eigenvalue calculations. We show the results in Fig. S1 (c-e) for a refractive index contrast of 0.2. We see that due to roughness, the cavity eigenmodes strongly mix with spurious eigenmodes for low values of the roughness, making it impossible to study the effect of roughness in these cavities through this method.

From these results, we argue that since the main effect of the loss of lateral confinement is the increase of the FSR of the cavity spectrum, we can use our study of smaller cavities as a representative indication of the dominant phenomenology in the very long 360 $\mu$m diode laser cavities. Furthermore, on the one hand we have shown in the main text that the analytical FP model, which stems essentially from a 1D cavity, captures well the phenomenology observed for cavities of dimensions $10 \mu$m $\times$ $30 \mu$m. Then, on the other hand, since the loss of lateral confinement in the very large aspect ratio diode lasers means that they will also behave as quasi-1D FP cavities, then we conclude that our analytical model and conclusions also apply to larger FP cavities which are beyond the current numerical capabilities.


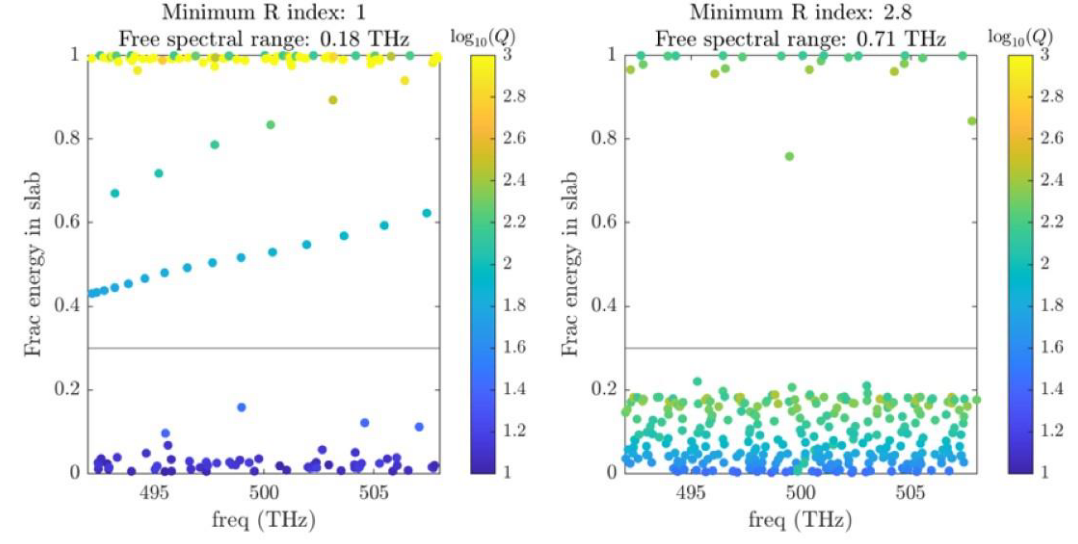


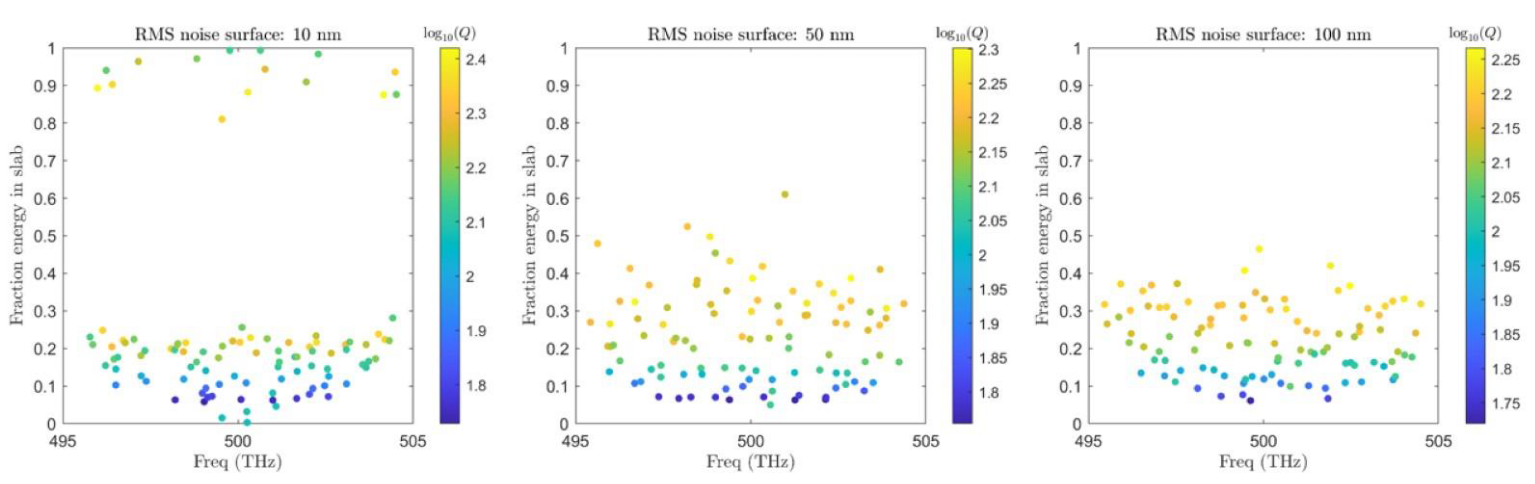


**Fig. S1** Fraction of the EM energy inside a cavity of $n_{bg}=3$ as a function of frequency. Colour scale represents the quality factor. a,b) Pristine case for refractive index contrast of 2 (a) and 0.2 (b), demonstrating that even for small refractive index contrast, the slab holds resonant modes, fewer of them (Hence the larger FSR). c-e) Refractive index contrast of 0.2 but with a rough boundary, demonstrating the inability of the numerical method to distinguish proper cavity modes with spurious modes.


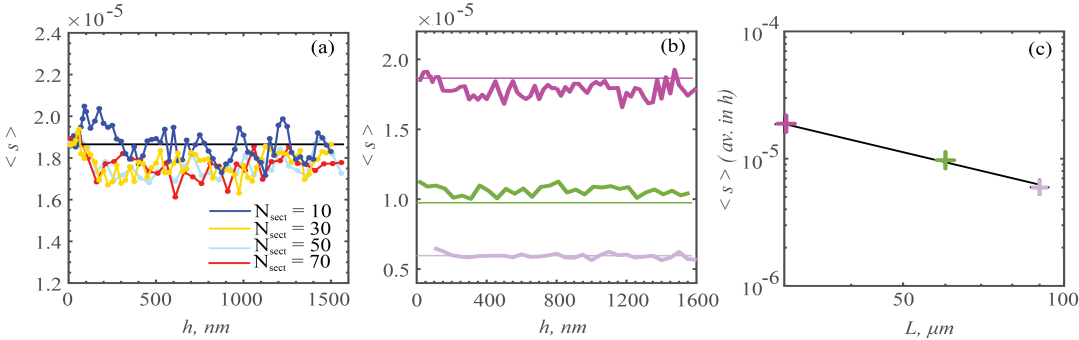


**Fig. S2** Dimensionless average spectral separation, ⟨*s*⟩, as a function roughness depth fluctuation, h, for different number of the sections *N*_sect_ for a FPC with a randomly corrugated wall. b. Dimensionless average spectral separation ⟨*s*⟩ as a function of roughness depth h for FPCs with various lengths *L*. Different coloured lines correspond to cavities of 30 (purple), 60 (green), and 90 (rose) $\mu$m respectively. Horizontal lines correspond to $\left\langle s \right\rangle$in the pristine case for the different cavity lengths. c. Mean (in $h$) average spectral separation as a function of FPC length in log-log scale. The black solid line represents the fit of the numerical data analytical expression ${5.62\times{10}^{-10}}/L$. *N*_sect_ = 40 for panels b and c.

1. Numerical inspection: TM polarization

In this section, we present the spectral characteristics of a Fabry-Perot cavity with a randomly crenelated wall for TM polarization.

**Fig. S2** presents the average spectral separation, ⟨*s*⟩, for transverse magnetic (TM) polarization as a function of geometric parameters of Fabry–Perot cavities with a randomly crenelated wall. These results are directly analogous to those shown for transverse electric (TE) polarization in the main text (Fig. 2) and demonstrate consistent behavior between the two polarizations.

In panel (a) of Fig. S2, ⟨*s*⟩ is shown as a function of roughness depth *h* for various numbers of sections, *N_sect_*, at fixed cavity length $L=30 \mu m$. As in the TE case, the values fluctuate around the horizontal solid line, which corresponds to the free spectral range (FSR) of the regular, non-disordered cavity.

Panel (b) shows ⟨s⟩ versus *h* for different cavity lengths ($L = 30, 60, 90 \mu m$) with *N_sect_* fixed at 40. The results again follow the same trend as in the TE case: for each *L*, the values fluctuate around the FSR of the corresponding regular cavity, shown by horizontal lines.

Since the dependence of ⟨*s*⟩ on *h* is weak, we average ⟨s⟩ over all *h* values and plot the result in panel (c) as a function of *L* on a log-log scale. The average spectral separation decreases with increasing cavity length. The black solid line shows the analytical expression $\langle s\rangle=(4.87\times10⁻¹⁰)/L$, which fits the numerical results (crosses) well over the entire range.

Thus, the results for TM polarization closely mirror those obtained for TE polarization.

**Fig. S3** presents the inverse quality factor, ⟨*Q*^–^¹⟩, for TM polarization as a function of structural disorder of the Fabry–Perot cavity wall. These results are directly compared to those reported in the main text for TE polarization (Fig. 3) and serve to confirm the polarization-independence of the observed spectral broadening trends.


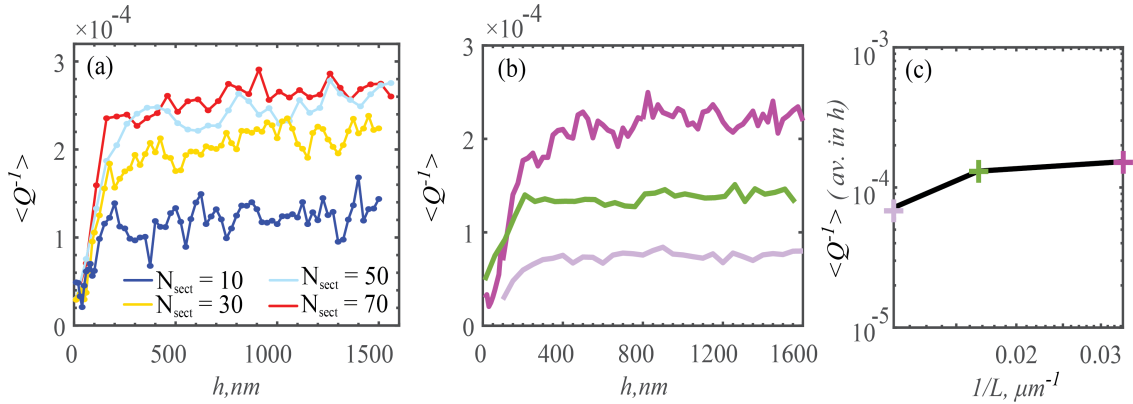


**Fig. S3** a. Dimensionless average spectral broadening ⟨*Q*^–^¹⟩ as a function of roughness depth, *h*, for different number of the sections *N*_sect_. Cavity length is 30 μm. **b.** Dimensionless average spectral broadening as a function of roughness depth for different FPC lengths, $L$**:** 30 μm (purple), 60 μm (green) and 90 μm (red) respectively. **c.** Spectral broadening, averaged over roughness depth (*h*), as a function of the inverse Fabry–Perot cavity length, shown on a log-log scale. *N*_sect_ = 40 for panels b and c.

Panel (a) shows ⟨*Q*^–^¹⟩ as a function of roughness depth *h* for several values of *N_sect_*, with the cavity length fixed at $L = 30 \mu m$. As in the TE case, ⟨*Q*^–^¹⟩ initially increases rapidly with *h* and then reaches a plateau. However, compared to the TE results, the TM curves exhibit a sharper increase and reach saturation at much smaller *h* values, particularly for higher *N_sect_* values. Additionally, the plateau values of ⟨*Q*^–^¹⟩ are marginally higher for TM polarization, suggesting a slightly stronger sensitivity to corrugation-induced broadening.

In panel (b), ⟨*Q*^–^¹⟩ is plotted as a function of *h* for cavities of different lengths (*L* = 30, 60, and 90 μm), with *N_sect_* fixed at 40. The results show the same general trend as observed for TE polarization: a sharp initial increase in ⟨*Q*^–^¹⟩ followed by saturation.

Panel (c) presents the average asymptotic value of ⟨*Q*⁻¹⟩ (i.e., averaged over large *h*) as a function of the inverse cavity length, 1/*L*, plotted on a log-log scale. The data follow a linear trend.

Thus, the results confirm that both TE and TM polarizations exhibit the same qualitative features. However, TM modes are marginally more susceptible to spectral broadening than TE modes, with slightly higher ⟨*Q*^–^¹⟩ values and sharper growth behavior.

1. First order perturbation theory for FPC TE modes

In this section we apply the first order perturbation theory developed in [2] to FPC eigenmodes to study how surface roughness modifies the system’s eigenfrequencies. In our implementation, the noise profile imposed on an edge of width $W$ is given by

$\begin{aligned} h\left( x \right)=\sigma_{h}\sum_{n=1}^{N_{max}} \frac{\mathcal{N}_{n}}{\sqrt{\frac{1}{2}\sum_{n=1}^{N_{max}} \left( \frac{\mathcal{N}_{n}}{n^{\beta}} \right)^{2}}n^{\beta}}\cos\left( 2\pi n\frac{1}{2}\left( \frac{x}{\frac{W}{2}}+1 \right)+U_{n} \right).\#\left( S17 \right) \end{aligned}$Where $\mathcal{N}_{n}$ is a random number extracted from a normal distribution with mean 0 and standard deviation 1, and $U_{n}$ is a random number extracted from a uniform distribution between 0 and $2\pi$. In the expression above, $x\in\frac{W}{2}\left[ -1,1 \right]$. This noise profile modifies the y coordinate of the slab boundary, which in the pristine limit we say is found at $y=L/2$. The eigenfrequency modification due to roughness in the case of TE modes can be estimated through [1]

$$\begin{aligned} \frac{d\omega}{d\sigma_{h}}=-\frac{\omega^{\left( 0 \right)}}{2}\frac{\left\langle E^{\left( 0 \right)}\left| \frac{d\epsilon}{d\sigma_{h}} \right|E^{\left( 0 \right)} \right\rangle}{\left\langle E^{\left( 0 \right)}\left| \epsilon\right|E^{\left( 0 \right)} \right\rangle}=-\frac{\omega^{\left( 0 \right)}}{2}\frac{\int dx\frac{dh}{d\sigma_{h}}\left| E_{\parallel}^{\left( 0 \right)}\left( x,y=\frac{L}{2} \right) \right|^{2}}{\int dx\left| E_{\parallel}^{\left( 0 \right)}\left( x,y=\frac{L}{2} \right) \right|^{2}}, \#\left( S18 \right) \end{aligned}$$

where $E_{\parallel}^{\left( 0 \right)}$correspond to the fields parallel to the material boundary in the pristine case. In what follows, we take two complementary approaches to predict the eigenfrequency shift due to roughness: First, we approximate the FPC modes by those of a rectangular slab bound by a perfect conductor, and then, we apply the perturbation theory to numerically obtained slab eigenfunctions.

**Approximated analytical FPC modes**

We now attempt to mimic the eigenmodes of the cavity FPC by using instead the modes of a perfect electric conductor (PEC) cavity. In reality, dielectric contrast provides the confinement necessary for the optical modes, and these modes spill out of the dielectric slab. Nevertheless, the actual FPC modes look very similar to those of a PEC cavity. To mimic this spill-out of the modes, we will instead look for the modes of a PEC slab that is slightly larger than the actual cavity, say of width: $W+\delta_{W}$, and length $L+\delta_{L}$. The optical modes for such cavity are given by:

$$\begin{aligned} E_{NM}^{\left( 0 \right)}=A_{NM}\sin\left( N\pi\left( \frac{x}{W+\delta_{W}}+\frac{1}{2} \right) \right)\sin\left( M\pi\left( \frac{y}{\left( L+\delta_{L} \right)}+\frac{1}{2} \right) \right),\#\left( S19 \right) \end{aligned}$$

where $N$ and $M$ are integers, and $A_{NM}$ is a normalization factor. Introducing these expressions into Eq. (S18), together with the roughness parametrization, and carefully taking the limit $\delta_{W}/W\to0$, it can be shown that within first order perturbation theory, the eigenfrequencies are modified according to

$$\frac{1}{\omega_{NM}^{\left( 0 \right)}}\frac{d\omega_{NM}^{\left( 0 \right)}}{d\sigma_{h}}=-\frac{1}{2\pi}\sum_{n=1}^{N_{max}} \frac{\mathcal{N}_{n}}{n^{\beta}\sqrt{\frac{1}{2}\sum_{n^{'}=1}^{N_{max}} \left( \frac{\mathcal{N}_{n^{'}}}{{n^{'}}^{\beta}} \right)^{2}}}\int_{0}^{2\pi} dz \cos\left( nz+U_{n} \right){\sin\left( \frac{N}{2}z \right)}^{2}=$$

$$\begin{aligned} =\frac{1}{4}\sum_{n=1}^{N_{max}} \frac{\mathcal{N}_{n}\cos\left( U_{n} \right)\delta_{N,n}}{n^{\beta}\sqrt{\frac{1}{2}\sum_{n^{'}=1}^{N_{max}} \left( \frac{\mathcal{N}_{n^{'}}}{{n^{'}}^{\beta}} \right)^{2}}}=\frac{\mathcal{N}_{N}\cos\left( U_{N} \right)}{4N^{\beta}\sqrt{\frac{1}{2}\sum_{n^{'}=1}^{N_{max}} \left( \frac{\mathcal{N}_{n^{'}}}{{n^{'}}^{\beta}} \right)^{2}}}.\#\left( S20 \right) \end{aligned}$$

This expression shows that for normally distributed roughness, in a given random noise realization the eigenfrequency change may be either positive or negative, depending on the actual outcomes of $\mathcal{N}_{N}$, and $U_{N}$. Nevertheless, one can see that across different noise realizations, the expectation value of this quantity becomes:

$$\begin{aligned} \left\langle\frac{1}{\omega_{NM}^{\left( 0 \right)}}\frac{d\omega_{NM}}{d\sigma_{h}} \right\rangle=0 \#\left( S21 \right) \end{aligned}$$

and therefore, to leading order and on average, modes are expected to remain at similar eigenfrequencies, regardless of the noise amplitude.

**Numerical testing**

To further test this result, we now apply Eq. S18 to the numerically determined eigenmodes of a 3 by 10 μm FPC. To do so, we obtain 300 eigenmodes of the pristine FPC, and evaluate Eq. S18 by generating random noise realizations according to Eq. S17. In **Fig. S4** we show the average predicted frequency shift as a function of the number of random realizations, demonstrating that the expectation value of the frequency shift vanishes too for the numerically determined eigenmodes.

These results are consistent with what is observed in Fig. 5a and Fig. 6a, where for $\sigma_{h}\in[{10}^{-2},{10}^{-1}]$ the resonant eigenfrequencies remain stable, while the quality factors of the modes quickly degrade, and explains why our simplified single-mode analytical model is able of capturing the relevant physics.


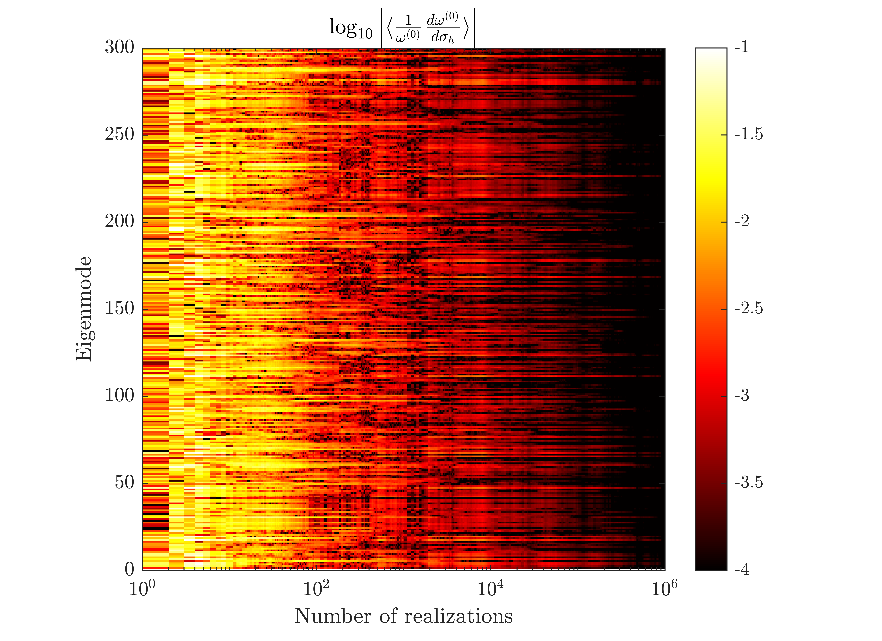


**Fig. S4** Average over realizations of predicted frequency shift of the eigenmodes of a pristine 3 by 10 micrometers FPC.

1. Analytic estimation of free spectral range:

In this section we apply the concept of DOS to calculate the average free spectral range in our 2D cavities. We assume that the walls of the cavity of dimensions *L*×*W* are made of perfect electric conductor (PEC), and thus the momentum values attainable are given by: $k_{x}=N\pi/L$ and $k_{y}=M\pi/W.$ This gives an area per mode in momentum space of $\Omega=\frac{\pi^{2}}{LW}=\pi^{2}/A$. For a 2D system, the number of modes with momentum between $k_{1}$ and $k_{2}$ can be calculated through:

$$\begin{aligned} N=\frac{1}{4} \frac{A}{\pi^{2}}\int_{k_{1}}^{k_{2}} dk 2\pi k=\frac{A}{4\pi}\left( k_{2}-k_{1} \right)\left( k_{2}+k_{1} \right) \#\left( S22 \right) \end{aligned}$$

Where we have divided by 4 to consider that only positive momenta contribute to the total number of modes in our PEC cavity. Now, say that we want to find the modes that occur around a centre momentum value $k_{c}$, and in an annular region of total width $\delta k$. Then we can approximate $k_{1}=k_{c}-\delta k/2$ and $k_{2}=k_{c}+\delta k/2$, and so, the total number of modes in this region is given by:

$$\begin{aligned} N=\frac{Ak_{c}}{2\pi}\delta k \#\left( S23 \right) \end{aligned}$$

Now we focus our attention on the calculation of the dimensionless spectral spacing. In our simulations, we look for $N_{p}$ eigenvalues closest to the eigenfrequency $\nu_{c}$, and calculate this magnitude as

$$\begin{aligned} \left\langle s \right\rangle=\frac{1}{N_{p}-1}\sum_{n=1}^{N_{p}-1} \frac{\nu_{n+1}-\nu_{n}}{\bar{\nu}} \#\left( S24 \right) \end{aligned}$$

which we can approximate here as

$$\begin{aligned} \left\langle s \right\rangle\approx\frac{1}{\nu_{c}}\frac{1}{N_{p}-1}\sum_{n=1}^{N_{p}-1} \left( \nu_{n+1}-\nu_{n} \right)=\frac{1}{N_{p}-1}\frac{\left( \nu_{N_{p}}-\nu_{1} \right)}{\nu_{0}} \#\left( S25 \right) \end{aligned}$$

by noting that all frequencies remain close to the central frequency of the search. Note also that this expression only depends now on the largest and smallest frequencies found among those closest to $\nu_{c}$. We can draw a parallel between these two calculations by changing into momentum and making the relations:

$$\begin{aligned} \nu_{c}=\frac{k_{c}c}{2\pi} \\ \nu_{N_{p}}=\nu_{c}+\frac{\delta\nu}{2}=\frac{k_{c}c}{2\pi}+\frac{1}{2}\delta k\frac{c}{2\pi} \\ \nu_{1}=\nu_{c}-\frac{\delta\nu}{2}=\frac{k_{c}c}{2\pi}-\frac{1}{2}\delta k\frac{c}{2\pi} \#\left( S26 \right) \end{aligned}$$

which upon insertion give

$$\begin{aligned} \left\langle s \right\rangle\approx\frac{1}{N_{p}-1}\frac{\delta k}{k_{c}} \#\left( S27 \right) \end{aligned}$$

If now we equate the momentum spread in this expression, with the momentum spread needed to obtain $N_{p}$ modes in the expression coming from the analytic DOS calculation, we find:

$$\begin{aligned} \left\langle s \right\rangle\approx\frac{N_{p}}{N_{p}-1}\frac{2\pi}{Ak_{c}^{2}}\#\left( S28 \right) \end{aligned}$$

This expression indicates that for a sufficiently large number of sampled eigenmodes, the free spectral range is expected to evolve as:

$$\begin{aligned} \left\langle s \right\rangle\approx\frac{2\pi}{\left( k_{c}W \right)\left( k_{c}L \right)} \#\left( S29 \right) \end{aligned}$$

This gives an analytical dependence of 1*/L* of the FSR, and also gives an order of magnitude to the slope that one could expect in the figures of the main text.

To test this expression, we numerically evaluate the FSR by looking for 100 modes around a certain frequency interval (500 THz $\to k_{c}=\frac{n_{bg}2\pi\nu}{c}$) for different PEC cavity geometries and compare the obtained values with the analytical prediction. The result is:


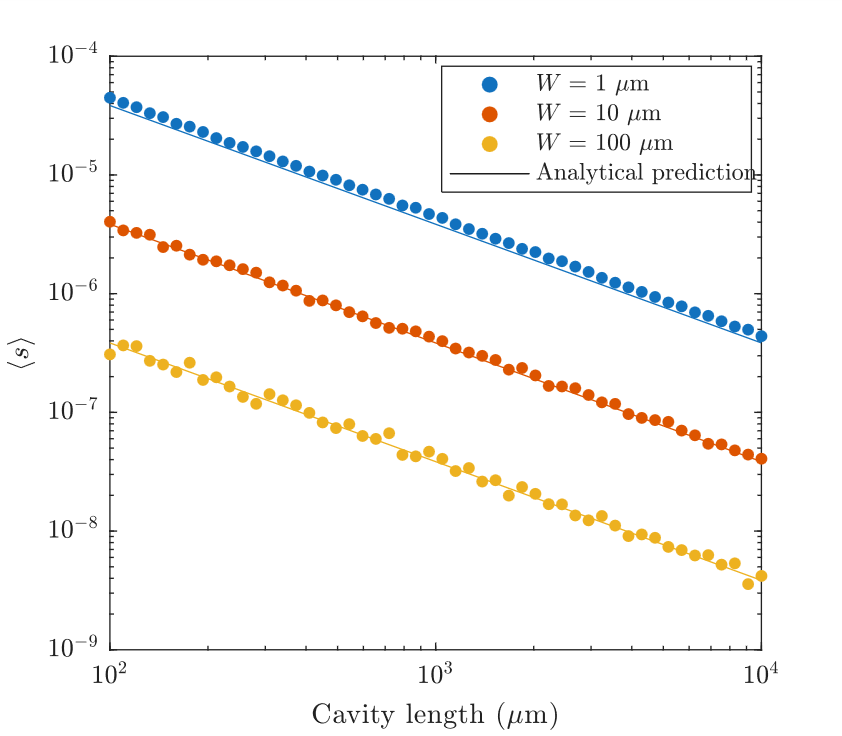


**Fig. S5.** Average over realizations of predicted frequency shift of the eigenmodes of a pristine 3 by 10 micrometers FPC.

Showing very good agreement between the analytical prediction and extracted values. We can now use this to compare with the non-ideal FP cavity in the main text and the extracted dependence of the FSR.

1. Comparison of uniform and normal random distribution for FPC wall roughness description

Figure S6 compares the results obtained using **normal** and **uniform random distributions** to describe the FPC wall roughness. Panels (a,b) correspond to the normal distribution, while panels (c,d) show the uniform case. In both cases, the dimensionless averaged spectral separation ⟨s⟩ remains nearly constant with increasing roughness amplitude *h*, exhibiting only small fluctuations around the mean value. The averaged linewidth broadening ⟨*Q*⁻¹⟩ shows a gradual increase with *h*, followed by a tendency toward saturation at large depths. These common trends are consistently observed for different numbers of roughness sections, indicating similar statistical behavior for the two distributions.


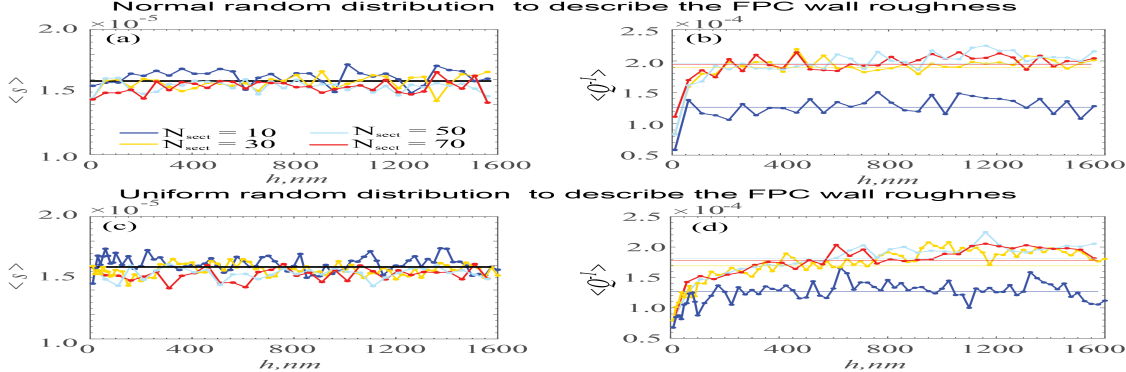


**Fig. S6** Comparison between the normal and uniform random distributions used to describe the FPC wall roughness. The results are shown for a cavity with width *W =*10 μm and length *L =*30 μm. Panels (a,b) show results obtained with the normal random distribution, and panels (c,d) correspond to the uniform random distribution. The plots present the dimensionless averaged spectral separation, (a,c), and averaged spectral broadening, (b,d), as functions of the roughness depth for different section numbers. Horizontal black line in panels (a,c) correspond to $\left\langle s \right\rangle$in the pristine case. Horizontal lines in panels (b,d) correspond to mean values of ⟨*Q*^–^¹⟩.

1. Influence of Random Realization and Wall Generation Procedure

To verify that the results shown in the main text are not specific to a particular random realization of the wall roughness or to the method used to vary its amplitude, we performed additional tests.


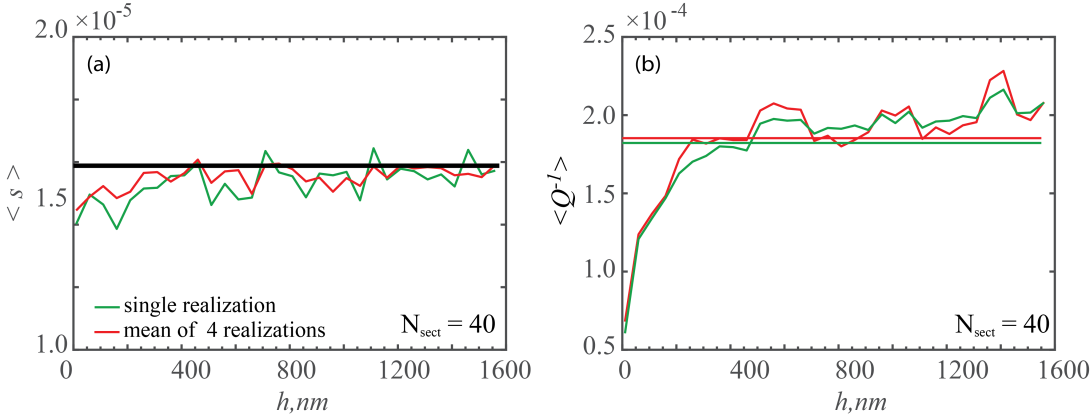


**Fig. S7** Comparison between the results obtained for a **single random realization** of the wall roughness (green) and the **average over four independent realizations** (red). Panels show the dimensionless averaged spectral spacing *⟨s⟩* (a) and avarage broadening *⟨Q⁻¹⟩* (b) as functions of roughness depth *h* for *N_sect_=*40. The results are shown for a cavity with width *W=*10 μm and length *L=*30 μm.

Figure S7 compares the results obtained for a single random realization of the wall roughness and the average over four independent realizations. Both the averaged spectral spacing ⟨s⟩ and linewidth broadening ⟨Q⁻¹⟩ exhibit nearly identical dependencies on the roughness depth *h*, indicating that a single realization provides a representative description of the average behavior.

To further assess the robustness of the results, Figure S8 compares two wall-generation schemes: in the reshaping case, a new random wall configuration was generated for each *h*, while in the non-reshaping case, the same wall shape was kept and its depth was gradually increased. The obtained curves show very similar trends, confirming that the specific method of varying the roughness amplitude has little effect on the averaged spectral characteristics.


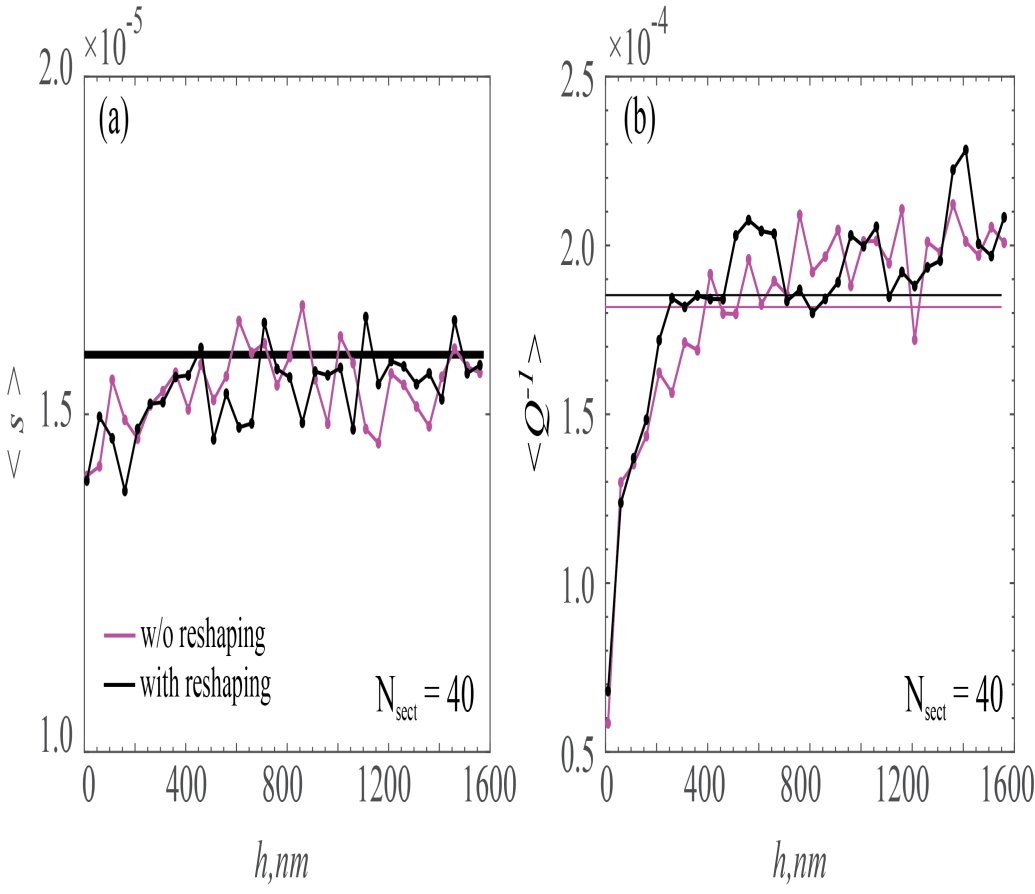


**Fig. S8** Comparison of the results obtained **with** and **without reshaping** the random wall profile for each roughness depth *h*. In the reshaping case (black), a new random wall configuration was generated for every value of *h*; in the non-reshaping case (magenta), the same wall shape was kept while its depth was gradually increased. Panels (a) and (b) show the dimensionless averaged spectral separation ⟨*s*⟩ and spectral broadening ⟨*Q*⁻¹⟩, respectively, for *N_sect_=*40. The results are shown for a cavity with width *W=*10 μm and length *L=*30 μm.

References

[1] H. Davies, ‘The reflection of electromagnetic waves from a rough surface’, *Proceedings of the IEE - Part IV: Institution Monographs*, vol. 101, no. 7, pp. 209–214, Aug. 1954, doi: 10.1049/pi-4.1954.0025.

[2] S. G. Johnson, M. Ibanescu, M. A. Skorobogatiy, O. Weisberg, J. D. Joannopoulos, and Y. Fink, ‘Perturbation theory for Maxwell’s equations with shifting material boundaries’, *Phys. Rev. E*, vol. 65, no. 6, p. 066611, June 2002, doi: 10.1103/PhysRevE.65.066611.
